# Keystone Veterinary Medical Association

**Published:** 1893-01

**Authors:** W. S. Kooker

**Affiliations:** Secretary


### SOCIETY PROCEEDINGS.

*Keystone Veterinary Medical Association.*—The regular meeting of the Keystone Veterinary Medical Association was held Nov. 11th, 1892. The meeting was called to order by the President, Dr. R. G. Webster. There answered roll call Drs. Miller, Hoskins, Raynor, Kooker, Bridge, Webster, Werntz, Sellers and Nunan. Minutes of previous meeting were read and approved. The committee on short term colleges reported the following :

*Whereas*, It has come to our notice that a number of those connected with the staff of the Bureau of Animal Industry propose establishing a Veterinary School in Washington, and

*Whereas*, All prominent veterinarians agree that it is absolutely necessary for intelligent and well-prepared young men to study at least three years to fit themselves to meet the multitudinous and intricate duties of the veterinary profession, and all progressive schools of this country now require three years' attendance, or propose inaugurating three-year courses of study during the coming session, and

*Whereas*, All veterinary schools of Europe require at least three years' attendance, and some of them four and five years, and the Royal College of Veterinary Surgeons of England has recently adopted a four-year plan of study, and the United States Veterinary Medical Association, at its recent meeting in Boston (Sept. 21st, 1892), declared in favor of a three years' course of study, and made this a necessary qualification for future membership in the Association, thus clearly showing the position of the best element of the veterinary profession, and

*Whereas*, We had a just right to expect from those who have been strong members of Veterinary Associations, and connected with a department that has ofttimes realized the incomplete education of many of its representatives, to have given all of its strong influence to elevate the veterinary profession of the land, and not to retard its progress by establishing and maintaining a new two-year short term Veterinary School: Be it

*Resolved*, That this Society recognize the utter impossibility of imparting sufficient veterinary instruction to students in two college sessions to enable them to practice Veterinary Medicine and Surgery intelligently, and that it regards the establishment and maintenance of schools giving such course of instruction as a menace to the progress of Scientific Veterinary Medicine. Be it further

*Resolved*, That this Association do urge upon those who are to establish this new veterinary college in Washington requiring but two sessions attendance upon instruction, to reconsider their action and to refrain from doing that which will tend to lower the standard of the veterinary profession, retard the progress of Veterinary Education, and lessen the dignity and standing of graduates of American Veterinary Colleges in this and foreign countries. Be it further

*Resolved*, That a copy of these resolutions be sent to the Secretary of the United

ARMY MEDICAL LIBRARY WASHINGTON, D. C. U. S. A.

States Veterinary Medical Association, the President of said College, and to each of the veterinary journals.

W. Horace Hoskins,

A. T. Sellers,

J. B. Raynor, } *Committee.*

*Whereas*, We, the members of the Keystone Veterinary Medical Association, have noticed with a great degree of pleasure the action of the Board of Trustees and Faculty of the American Veterinary College, in their announcement that on and after 1893 they will adopt an obligatory three years' course. We recognize this movement on the part of the above school as a wise one, and we congratulate them on the wisdom they have displayed in recognizing the greater needs of the members of the veterinary profession, and we trust that their example will be followed by all other two-year schools of our country, and we hereby express our disapproval of all schools whose term embraces but two short college years, and who, in addition, offer other inducements that tend to lower the standard of veterinary medicine in this country, and we trust that the time is not far distant when other associations, local and State, shall follow in the footsteps of the precedent about to be established by the U. S. V. M. A., and will require from their applicants for membership, the same conditions as will be demanded in the parent association. We recognize that the time has arrived when no two years school of less than six months each, can properly equip young men for the future duties of the veterinary profession of this country.

W. Horace Hoskins,

J. B. Raynor,

A. T. Sellers, } *Committee.*

On motion the report was accepted with thanks to the committee. Dr. W. B. E. Miller read a paper on "Animal Castration." It was thorough in its details of the operation in all its complications, and replete with statistics showing his percentage of deaths and the causes thereof. On a motion by Dr. Raynor, seconded by Dr. Sellers, a vote of thanks was tendered Dr. Miller for his excellent paper. The remarks on the paper resulted in drifting to the subject of Tetanus, which was very fully discussed by all present. Dr. Raynor cited a very interesting case.

W. S. Kooker, *Secretary.*

